# Autumnal Wisdom

**Published:** 1872-11

**Authors:** 


### AUTUMNAL WISDOM.

HOWEVER warm an occasional day may be, take care not to change any garment for a lighter one. If you have to walk to your place of business, and fires are not yet begun to be lighted, avoid removing hat and overcoat for ten or fifteen minutes, so as to allow the extra bodily warmth, engendered by the walk, to fall to the natural heat; then take off the hat; next, in a few minutes, the overcoat. If the shoes or boots are changed, those about to be put on should be warmed on the inside, unless lined with woolen.

Professional men and bookkeepers acquire a habit of cold feet by having their feet rest on the wooden floor; a woolen mat should be always used, and it will be found to be a very great comfort.

Many families who have been to the country for health, have imbibed the seeds of intermittent fever, which are made to fructify by the chilliness occasioned in the mornings, in the wish to put off for a little while longer the trouble and dirt and dust connected with lighting fires, with the result of having a physician come to the house half the winter, trying to get the malaria, or rather miasmatic poisons, out of the systems of the children. From the first day of October, a blazing fire should be kindled at sunrise and sunset, in the family room, and by very instinct it will be found that all the children will habitually gather there, as well as grandmother and grandfather.

The editor knew a most estimable gentleman of sixty, who habitually sat for an hour or two every morning in the fall of the year, with a heavy blanket over his legs, to keep him warm; he did it to accommodate his most excellent and tidy wife, who did "so hate to have the parlor carpet covered with ashes."

Get into the habit, before cold winter comes, of closing the mouth and breathing through the nose in going out of doors, so as to warm the air in its long passage through the head towards the lungs.

When you first come into the house for the night, pull off the shoes worn through the day, and put the feet before the fire for a few minutes; then slip them into warm slippers; there will be a comfortableness in the feeling about the feet for the remainder of the evening, which will well repay the trouble. On the other hand, many a severe and troublesome cold has been taken by putting warm and perhaps perspiring feet into cold slippers, condensing the perspiration, and keeping the stockings damp until bedtime.

